# Supplementary material for: Enzymatic conversion of human blood group A kidneys to universal blood group O
Source: Nat Commun. 2024 Mar 30;15:2795. doi: 10.1038/s41467-024-47131-9 (PMC10981661; doi:10.1038/s41467-024-47131-9)
Supplement: Supplementary file 1 — Supplementary Information [file 41467_2024_47131_MOESM1_ESM.pdf]

Enzymatic conversion of human blood group A kidneys to universal blood group O: Supplementary Information

1 Supplementary Table

Timepoint	Control						Treated					
	1		2		3		1		2		3	
	Count	MFI	Count	MFI	Count	MFI	Count	MFI	Count	MFI	Count	MFI
**Pre**	43311	45	37306	54.1	39579	74.1	43482	50	33055	61.9	37365	69.9
**0hr + Ab**	10879	5676	20811	2378	12684	1928	14649	6677	28606	1902	26896	2770
**5 min**	11016	5341	10007	2171	12588	2120	11789	5025	12872	2545	25153	2760
**10 min**	12273	3182	15188	1748	18871	1883	11387	3743	11037	2789	15596	2705
**15 min**	12477	3451	16810	1569	18105	1558	12797	6655	11881	2485	15498	2687
**20 min**	12711	2934	10697	1486	13799	1491	13243	6791	10917	2331	24082	2687
**25 min**	14982	2571	18892	1412	12765	1417	12368	6700	10634	2723	23768	2714
**30 min**	13582	2485	13669	1234	14343	1342	13225	6283	21594	2696	14750	2615
**1hr**	16844	1814	16880	1130	15168	1074	11609	6791	10843	2687	23017	2571
**1.5hr**	17353	1713	23003	997	15654	973	11763	6954	19340	2395	14785	2435
**2hr**	17858	1569	19759	916	15955	808	11805	6412	25351	2387	14166	2519
**3hr**	22312	1307	14994	870	22270	703	11910	6240	21023	2354	12923	2411
**4hr**	23539	1126	25854	789	24163	618	10942	6177	10255	2395	13947	2370

Supplementary Table 1. Flow cytometry count and MFI statistics for RBCs incubated with perfusate during ABOi reperfusion of control and treated kidneys. Each column number refers to one biological pair of human kidneys. Gated RBC count and median fluorescence intensity (MFI) are indicated for each kidney at each specified timepoint. “Pre” refers to a perfusate sample taken immediately before antibody addition. “0hr + Ab” indicates immediately after antibody addition.

RBC – red blood cell; ABOi – ABO-incompatible.

2 Supplementary Figures

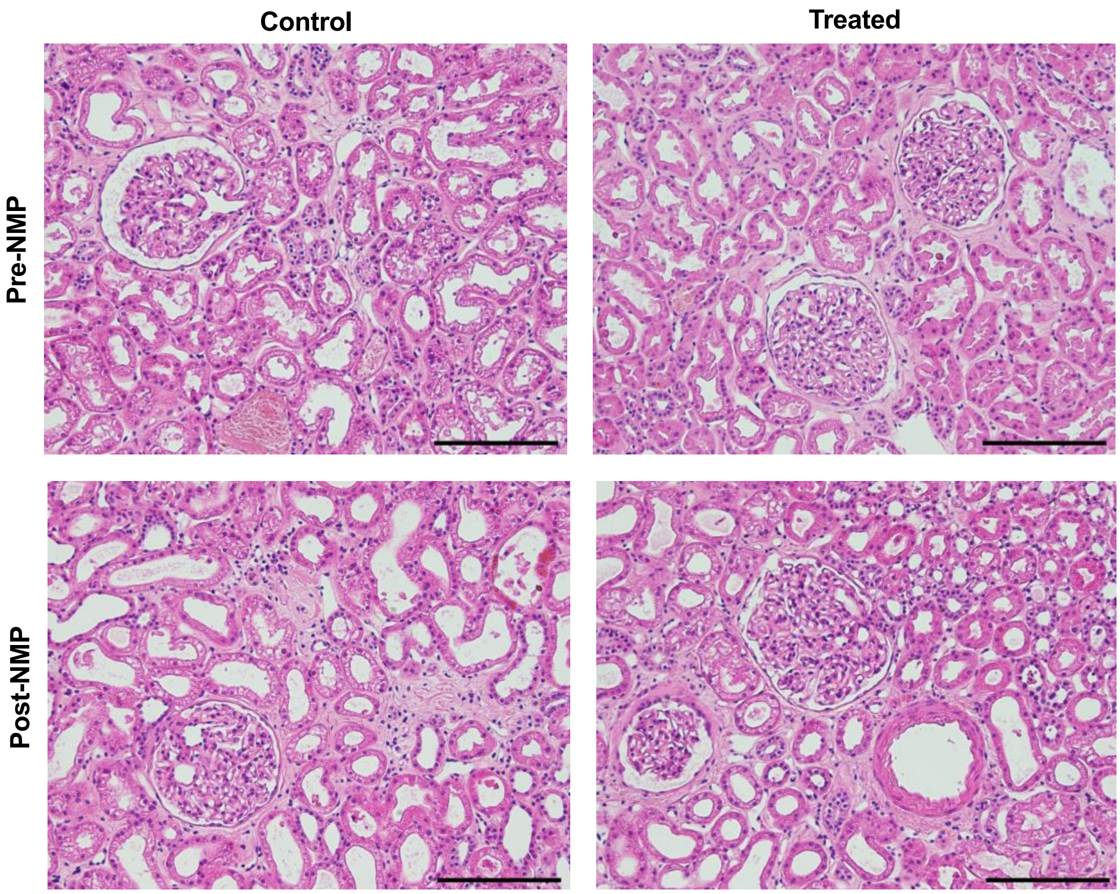

Supplementary Figure 1. Histology of kidneys after normothermic machine perfusion (NMP) in control and *Fp*GalNAc DeAc and *Fp*GalNase treated kidneys. Representative haematoxylin and eosin-stained kidney cortical biopsies from n=3 biological pairs taken before NMP (pre) and after 6hrs NMP (post) in control and treated kidneys. Scale bar represents 200µm.

NMP – normothermic machine perfusion.

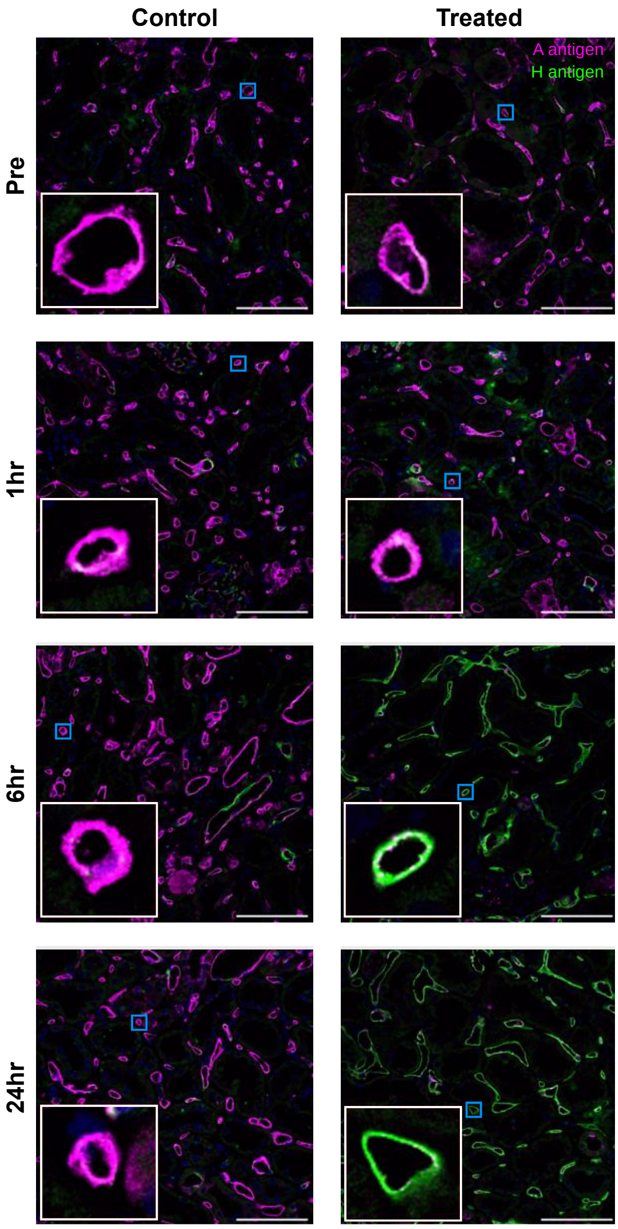

Supplementary Figure 2. Immunofluorescence staining of kidney cortical biopsies from a biological pair of kidneys during 24hrs HMP where one kidney (treated) received treatment with *Fp*GalNAc deacetylase and *Fp*Galactosaminidase. At each timepoint (pre-treatment, 1hr, 6hrs or 24hrs after enzyme addition) a composite image of blood group A antigens (magenta) and H antigens (green) is shown. An 8x zoom inlay of a representative peritubular capillary (blue box) is shown in the bottom left of each panel (white box). Images are representative of n=3 biological pairs of kidneys. Scale bar represents 100µm. HMP – hypothermic machine perfusion.

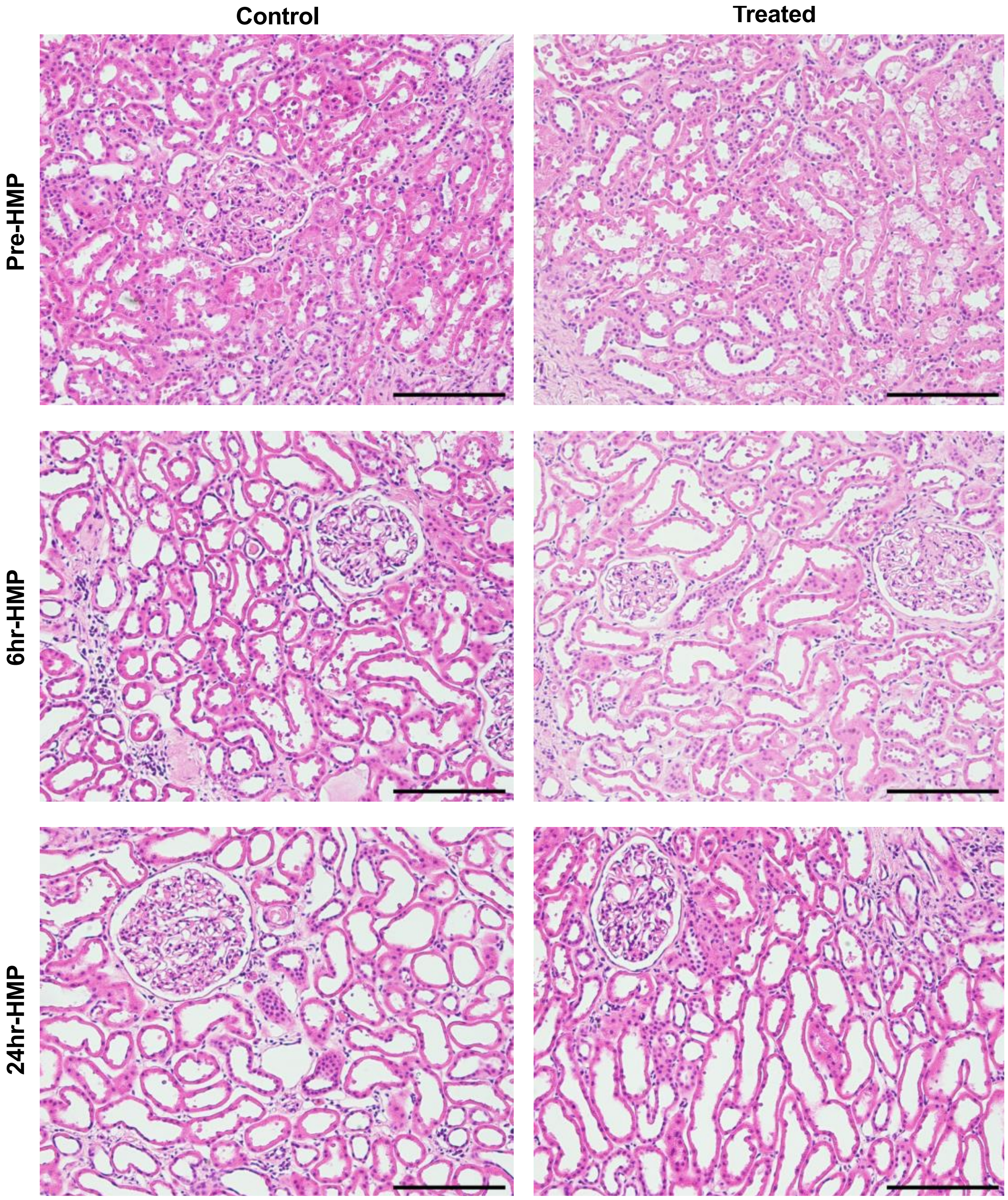

Supplementary Figure 3. Histology of kidneys after hypothermic machine perfusion (HMP) in control and *Fp*GalNAc DeAc and *Fp*GalNase treated kidneys. Representative haematoxylin and eosin-stained kidney cortical biopsies from n=3 biological pairs taken before HMP (pre) and after 6hrs and 24hrs HMP in control and treated kidneys. Scale bar represents 200µm.

HMP – hypothermic machine perfusion.

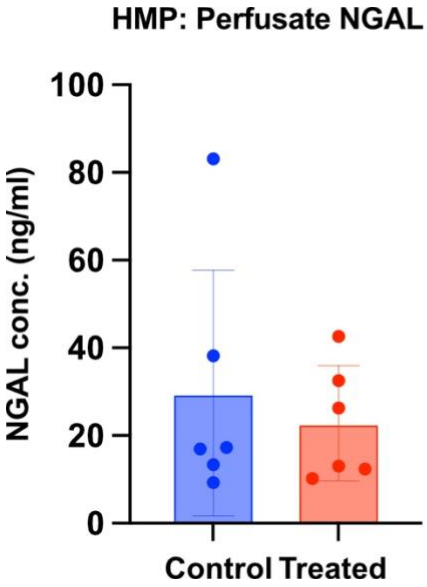

Supplementary Figure 4. NGAL concentration after 6hrs hypothermic machine perfusion (HMP) in control and *Fp*GalNAc DeAc and *Fp*GalNase treated kidneys. NGAL concentration in the circulating perfusate of control and treated kidneys after 6hrs HMP. Data are from 6hr perfusate samples from n=6 pairs (HMP cohort and HMP-ABOi cohort, combined). A two-tailed Wilcoxon matched-pairs signed rank test was used to compare control and treated groups ($p = 0.5625$). Error bars show mean ± standard deviation.

HMP – hypothermic machine perfusion.

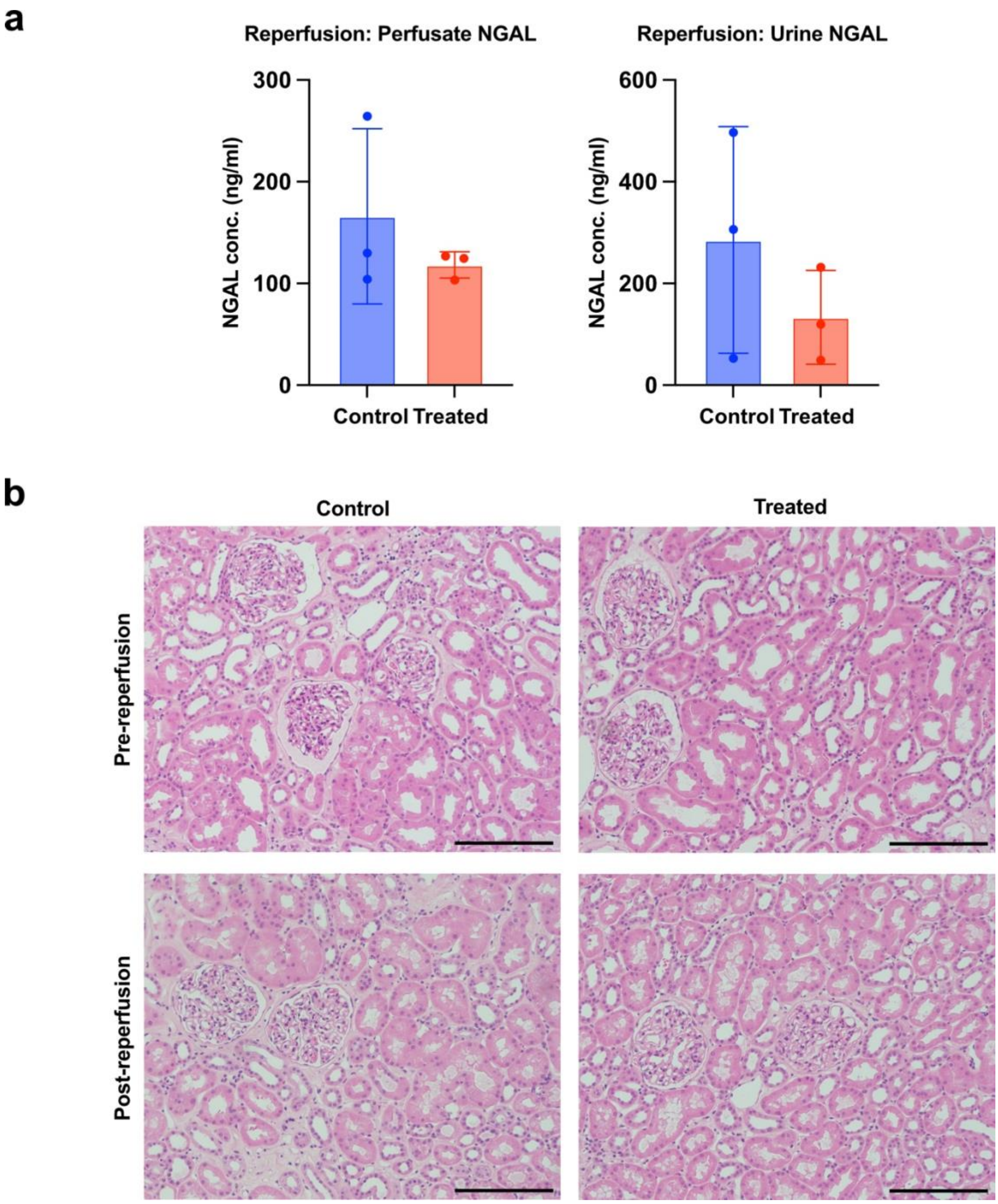

Supplementary Figure 5. NGAL concentration and histology in control and *Fp*GalNAc DeAc and *Fp*GalNase treated kidneys after ABOi reperfusion. (a) NGAL concentration in the perfusate and urine of control and treated kidneys after 4hrs ABOi reperfusion for n=3 biological pairs of human kidneys. A two-tailed Wilcoxon matched-pairs signed rank test was used to compare control vs treated groups (perfusate: $p = 0.5000$; urine: $p = 0.2500$). Error bars show mean ± standard deviation. (b) Representative haematoxylin and eosin-stained kidney cortical biopsies from n=3 biological pairs taken before ABOi-reperfusion (pre) and after 4hrs reperfusion (post) in control and treated kidneys. Scale bar represents 200μm. ABOi – ABO-incompatible.

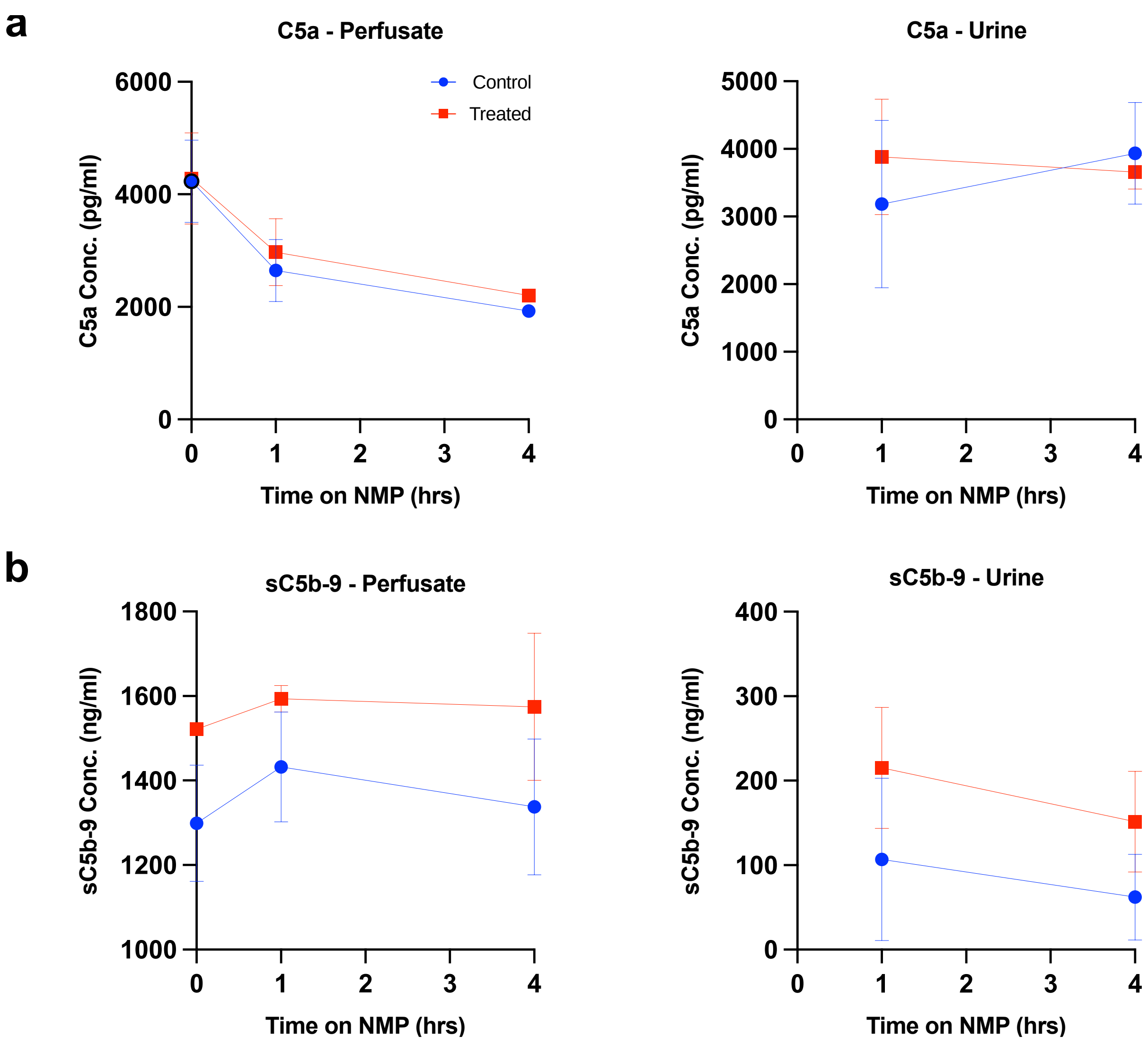

Supplementary Figure 6. C5a and sC5b-9 concentration during ABOi reperfusion. (a) Concentration of C5a in perfusate and urine samples from n=3 pairs of human kidneys during ABOi reperfusion. Perfusate samples were taken at the start of reperfusion (0hr) and at 1hr and 4hrs. Urine samples were taken at 1hr and 4hrs. (b) Concentration of soluble C5b-9 (sC5b-9) during reperfusion in the perfusate and urine from n=3 pairs of kidneys. All timepoints were the same as C5a. In all cases, control samples are shown in blue circles, and treated samples in red squares. A two-tailed Wilcoxon matched-pairs signed rank test with Holm-Sidak multiple comparisons correction was performed at each timepoint, where $p>0.05$ in all cases. Error bars represent mean ± standard deviation.

ABOi - ABO-incompatible; NMP – normothermic machine perfusion.

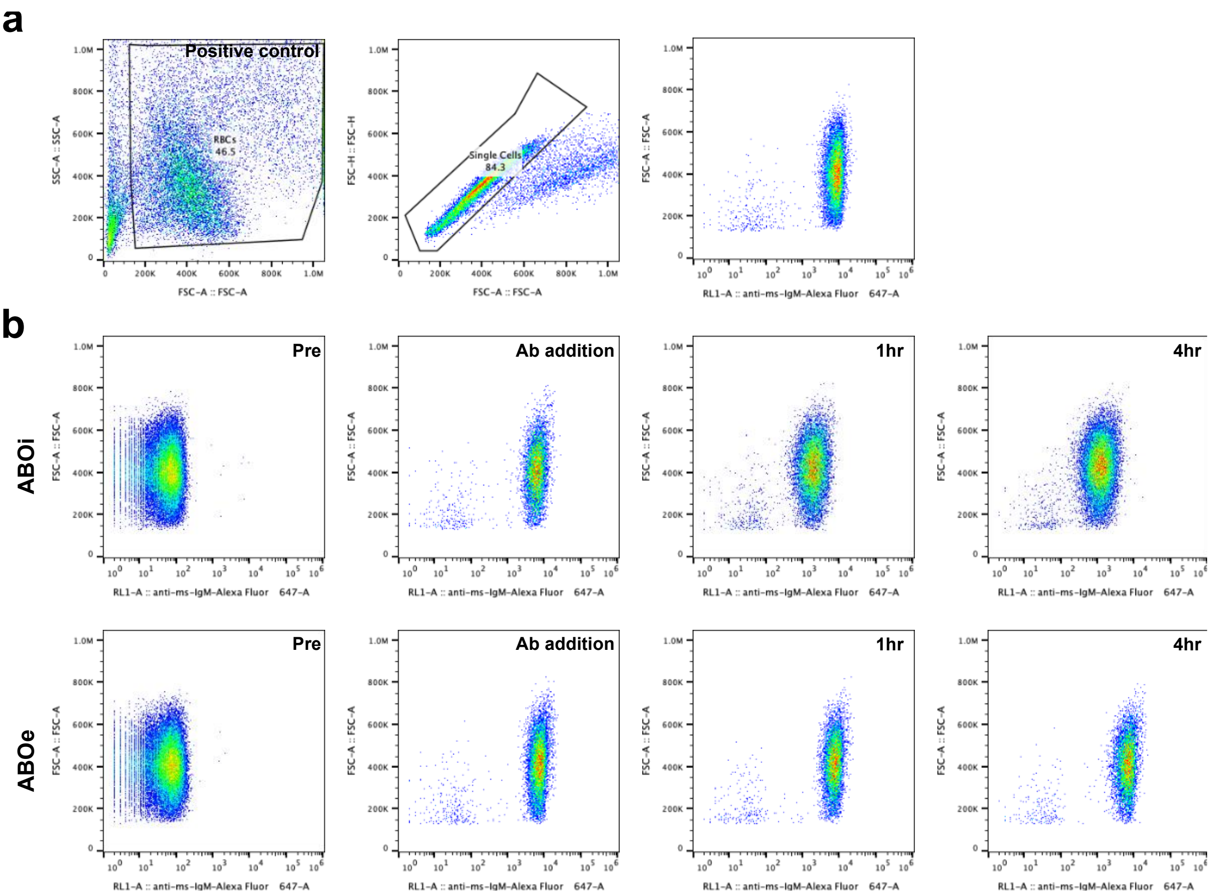

Supplementary Figure 7. Flow cytometry gating strategy for blood group A red blood cells incubated with reperfusion perfusate containing circulating antibodies. (a) Gating strategy for the positive control of blood group A RBCs incubated with the same antibody as added to the reperfusion perfusate solution (1:128) followed by anti-mouse IgM-AF647 for flow cytometric detection on RL1-A (excitation: 637nm; emission: 670/14nm). Single cells were gated on a graph of FSC-H/FSC-A. (b) Single cell gated RBC dot plots for FSC-A/RL1-A (anti-mouse IgM-AF647) for a representative pair of blood group A kidneys during reperfusion. One kidney (ABOi) was a control while the other kidney (ABOe) was treated with *Fp*GalNAc DeAc and *Fp*GalNase during HMP. Timepoints are indicated in the top right corner of each plot, where 'Pre' indicates perfusate samples taken prior to antibody addition; 'Ab addition' indicates perfusate samples taken immediately after antibody addition; '1hr and 4hr' indicate perfusate samples taken at the relevant timepoints post antibody addition.

RBCs – red blood cells; RL1-A – RL1 channel area; FSC-H – forward scatter height; FSC-A – forward scatter area; ABOi – ABO-incompatible; ABOe – enzyme-treated; HMP – hypothermic machine perfusion.

3 Supplementary methods

3.1 Acellular normothermic machine perfusion (NMP cohort)

Perfusate composition:

Ringer's solution	340ml
Human serum albumin (20%)	125ml
Dexamethasone	6mg
Calcium gluconate (10%)	10ml
Sodium bicarbonate (8.4%)	27ml

Supplements and infusions:

Glucose (5%)	3ml/hr
Prostacyclin (Epoprostenol; 0.5mg in saline)	3ml/hr
Synthamin 17 (10%) with	10ml/hr

- 15ml Sodium bicarbonate (8.4%)
- 5ml multivitamins
- 100iu insulin

Ringer's solution to replace urine output ml for ml

Protocol:

Three pairs of kidneys (NMP cohort) were perfused for 6hrs an oxygenated acellular perfusate at approx. 37°C using a modified method from Nicholson and Hosgood[1]. Briefly, each pair of kidneys was retrieved at the donor centre, flushed, and stored in a preservation solution on ice for transport. After arrival at our centre, the kidneys were benched, and the renal artery and ureter were cannulated. Kidneys flushed once more with 500ml Ringer's solution vis the arterial cannula. The kidneys were then perfused at approximately 37°C with 500ml acellular perfusate (see above) using an adapted paediatric cardiopulmonary bypass machine. After a stabilisation period of 20mins, the treated kidney of the pair was injected with 0.5ml of each of FpGalNAc deacetylase and FpGalactosaminidase, each at a concentration of 1mg/ml, via the arterial cannula (time 0hr). Renal blood flow (RBF) and mean arterial pressure (MAP) were recorded every 30mins. Biopsies were taken at the specified timepoints, and arterial perfusate, and urine samples were collected hourly. Total urine volume output per hour was also recorded. Each biopsy was split, with one tissue sample formalin fixed and paraffin embedded (FFPE) and the remaining sample flash frozen in liquid nitrogen before long-term storage at -80°C. All perfusate and urine samples were flash frozen in liquid nitrogen before long-term storage at -80°C.

3.2 Hypothermic machine perfusion

Protocol:

Six pairs of kidneys were perfused at approx. 4°C for 6hrs (n=3 pairs) or 24hrs (n=3 pairs) using a LifePort Kidney Transporter (Organ Recovery Systems, Itasca, IL, USA) as per the manufacturer's recommendations. Briefly, each pair of kidneys was retrieved and prepared as described for the NMP cohort without cannulation of the ureter. The kidneys were then immersed in a LifePort cassette containing 1L of Belzer MPS UW Machine Perfusion Solution and perfused for a 20 min stabilisation period before the treated kidney was injected with 1ml of each of FpGalNAc deacetylase and FpGalactosaminidase, each at a concentration of 1mg/ml, via the arterial cannula (time 0hr). The kidneys perfused for 6hrs had hourly RBF, MAP and temperature monitoring, alongside sampling and biopsies starting at 0hr. Kidneys perfused for 24hrs were monitored and sampled at the specified timepoints. Biopsies and samples of perfusion solution were also taken at these timepoints and stored as described for the NMP cohort.

3.3 Normothermic re-perfusion (ABOi cohort)

Perfusate composition:

Packed red blood cells (blood group O)	200ml
Human serum albumin (20%)	50ml
Ringer's solution	130ml
Human off-the-clot AB serum	50ml
Dexamethasone	3.3mg
Calcium gluconate (10%)	5ml
Sodium bicarbonate (8.4%)	27ml
Meropenem	500mg
Heparin	1500 IU
Creatinine	0.110g
Verapamil	2.5ml
Anti-A and anti-B (1:2048; 1:2048)	30.25ml

Supplements and infusions:

Glucose (5%)	3ml/hr
Prostacyclin (Epoprostenol; 0.5mg in saline)	3ml/hr
Synthamin 17 (10%) with	10ml/hr

- 15ml Sodium bicarbonate (8.4%)
- 5ml multivitamins
- 100iu insulin

Ringer's solution to replace urine output ml for ml

Monoclonal mouse IgM antibody (anti-A,B) titre was validated internally using microplate agglutination assays with 3% reagent A1 and B red blood cells (ALBAcyte A1 cells and B cells, Alpha Labs, Hampshire, UK)[2]. In antibody lots where the titre was less than 1:2048, protein concentration columns (Pierce™ Protein Concentrators PES, 100K MWCO; 88532; Thermo Fisher Scientific; MA, USA) were used to concentrate the antibody and titres were confirmed with microplate agglutination assays.

3.4 Image analysis

All images were analysed with FIJI software[3]. For quantification of antigen loss, a threshold of pixel intensity was determined on the anti-A channel based on the six images of the pre-treatment biopsy per experiment. The threshold was manually adjusted to include all vascular staining and a selection mask was made. This selected area was measured, and the integrated density value (a combination of fluorescence intensity and area) was recorded per image. This threshold was applied to all sections stained and imaged in the same experiment. These values were normalised to the mean pre-treatment value to determine percentage expression. Per biopsy, six randomly selected fields of view were measured and all data points per cohort were collated (n=3 kidneys per cohort, so 18 values per cohort). A similar procedure was used for *Ulex europaeus* (anti-H) staining, where the threshold was determined on the terminal biopsy of the enzyme treated group per experiment as this was considered to have maximal anti-H staining. For IgM staining quantification, the threshold value was determined on the 4hr ABOi control biopsy.

3.5 Flow cytometry

For fixation of red blood cells (RBCs), 40µl packed RBCs from a blood group A donor were washed in PBS followed by centrifugation at 600g for 5mins before resuspending in 1ml 0.05% glutaraldehyde solution in PBS. The cells were fixed for 10 mins at room temperature before centrifuging as before and washing twice in PBS + 0.6% BSA. Cells were resuspended in PBS + 0.6% BSA to form a 1% RBC solution.

To measure circulating antibody levels in the perfusate, 50µl of 1% fixed RBCS were pipetted in a 96-well U-bottomed plate. 50µl of PBS (unstained negative control; 2° only control), or perfusate sample (test) were added to the RBCs and incubated for 30 mins at RT while shaking on a microplate shaker. A positive control was included in each plate using anti-A-B as prepared above diluted in PBS to a final titre of 1:128. The plate was spun at 600g for 5 mins and the supernatant removed. RBC pellets were resuspended and washed three times in 200µl PBS + 0.6% BSA. After the final wash, the pellets were resuspended in PBS + 0.6% BSA containing goat anti-mouse$_{IgM}$-647 (A-21238; Invitrogen, Carlsbad, CA, USA) at a titre of 1:2000 and were incubated for 30mins at RT in the dark while shaking. The unstained control received only PBS + 0.6% BSA. The plate was centrifuged, and pellets washed as before. After the final wash, RBC pellets were resuspended in 250µl PBS + 0.6% BSA and kept in the dark.

For flow cytometry, the 96-well plate was analysed using an NXT Attune with Autosampler. RBCs and singlets were gated. Unstained controls were negative in all cases. At least 10,000 events were recorded per sample. Subsequent flow cytometry analysis was completed using FlowJo™ v10.9.0 Software (BD Life Sciences).

4 Supplementary References

1. Nicholson, M. L. & Hosgood, S. A. Renal Transplantation After Ex Vivo Normothermic Perfusion: The First Clinical Study. *American Journal of Transplantation* **13**, 1246–1252 (2013).
2. American Association of Blood Banks. *Technical Manual 20th Edition*.
3. Schindelin, J. *et al.* Fiji: an open-source platform for biological-image analysis. *Nat Methods* **9**, 676–682 (2012).
