# Supplementary material for: Enzymatic conversion of human blood group A kidneys to universal blood group O
Source: Nat Commun. 2024 Mar 30;15:2795. doi: 10.1038/s41467-024-47131-9 (PMC10981661; doi:10.1038/s41467-024-47131-9)
Supplement: Supplementary file 3 — Reporting Summary [file 41467_2024_47131_MOESM3_ESM.pdf]

nature portfolio

Corresponding author(s): Serena MacMillan

Last updated by author(s): Mar 10, 2024

Reporting Summary

Nature Portfolio wishes to improve the reproducibility of the work that we publish. This form provides structure for consistency and transparency in reporting. For further information on Nature Portfolio policies, see our Editorial Policies and the Editorial Policy Checklist.

Statistics

For all statistical analyses, confirm that the following items are present in the figure legend, table legend, main text, or Methods section.

n/a	Confirmed	
☐	☒	The exact sample size (*n*) for each experimental group/condition, given as a discrete number and unit of measurement
☐	☒	A statement on whether measurements were taken from distinct samples or whether the same sample was measured repeatedly
☐	☒	The statistical test(s) used AND whether they are one- or two-sided<br>*Only common tests should be described solely by name; describe more complex techniques in the Methods section.*
☒	☐	A description of all covariates tested
☐	☒	A description of any assumptions or corrections, such as tests of normality and adjustment for multiple comparisons
☐	☒	A full description of the statistical parameters including central tendency (e.g. means) or other basic estimates (e.g. regression coefficient) AND variation (e.g. standard deviation) or associated estimates of uncertainty (e.g. confidence intervals)
☐	☒	For null hypothesis testing, the test statistic (e.g. *F*, *t*, *r*) with confidence intervals, effect sizes, degrees of freedom and *P* value noted<br>*Give P values as exact values whenever suitable.*
☒	☐	For Bayesian analysis, information on the choice of priors and Markov chain Monte Carlo settings
☒	☐	For hierarchical and complex designs, identification of the appropriate level for tests and full reporting of outcomes
☒	☐	Estimates of effect sizes (e.g. Cohen's *d*, Pearson's *r*), indicating how they were calculated

Our web collection on statistics for biologists contains articles on many of the points above.

Software and code

Policy information about availability of computer code

Data collection	No code or other software was used to collect data for this study.
Data analysis	Flow cytometry analysis was completed with FlowJo (Version 10.9.0). Immunofluorescence images were analysed with Fiji (Version 2.9.0.) Statistical analysis was completed with GraphPad PRISM (Version 10.1.1). No other software packages or code were used in this study.

For manuscripts utilizing custom algorithms or software that are central to the research but not yet described in published literature, software must be made available to editors and reviewers. We strongly encourage code deposition in a community repository (e.g. GitHub). See the Nature Portfolio guidelines for submitting code & software for further information.

Data

Policy information about availability of data

All manuscripts must include a data availability statement. This statement should provide the following information, where applicable:

- Accession codes, unique identifiers, or web links for publicly available datasets
- A description of any restrictions on data availability
- For clinical datasets or third party data, please ensure that the statement adheres to our policy

The data produced and analysed in this study are described within the paper and Supplementary Information. Source data are provided as a Source Data file.

Research involving human participants, their data, or biological material

Policy information about studies with human participants or human data. See also policy information about sex, gender (identity/presentation), and sexual orientation and race, ethnicity and racism.

Reporting on sex and gender	Human donor kidneys were recruited blinded to sex. The donor demographics report the sex of the donors in Table 1.
Reporting on race, ethnicity, or other socially relevant groupings	N/A
Population characteristics	All donor characteristics are noted in Table 1.
Recruitment	Deceased human donor kidneys were accepted for this study after being declined for transplantation and subsequently offered for research. Kidneys were recruited based on (a) status as blood group A, and (b) as a biological pair.
Ethics oversight	This study has ethical approval was obtained from NRES: 15/NE/0408 and 22/WA/0167.

Note that full information on the approval of the study protocol must also be provided in the manuscript.

Field-specific reporting

Please select the one below that is the best fit for your research. If you are not sure, read the appropriate sections before making your selection.

☒ Life sciences ☐ Behavioural & social sciences ☐ Ecological, evolutionary & environmental sciences

For a reference copy of the document with all sections, see nature.com/documents/nr-reporting-summary-flat.pdf

Life sciences study design

All studies must disclose on these points even when the disclosure is negative.

Sample size	The sample sizes for this preliminary study were decided based on expected availability of appropriate kidney pairs with a minimum of n=3 per group.
Data exclusions	Due to sample availability, data from the 2.5hr and 3.5hr timepoints in the flow cytometry analysis of antibody levels during reperfusion were excluded due to a lack of appropriate replicates. All surrounding and remaining timepoints are detailed in the manuscript and this exclusion does not effect the conclusions drawn from this data.
Replication	Technical replicates were successful in all cases, with biological negative controls included in all appropriate experiments. Biochemical measurements were completed with technical duplicates. All image analysis involved six independent fields of view per biopsy for a total of 18 images per n=3 cohort. Biological replicates are detailed within the manuscript with a minimum of n=3 independent donor kidneys per group.
Randomization	Kidneys within each pair were randomly allocated to control or treatment conditions by a coin toss.
Blinding	Renal pathologist screening of H&E-stained biopsy material was blinded to the pathologist. Staining experiments were analysed blinded to the investigator, where study codes were used in lieu of control vs treated terminology. Kidney perfusion experiments were not blinded to treatment group due to the necessity of enzyme administration by the investigators. After data collection, numerical study codes were used to refer to each kidney for analysis.

Reporting for specific materials, systems and methods

We require information from authors about some types of materials, experimental systems and methods used in many studies. Here, indicate whether each material, system or method listed is relevant to your study. If you are not sure if a list item applies to your research, read the appropriate section before selecting a response.

Materials & experimental systems

n/a	Involved in the study	
☐	☒	Antibodies
☒	☐	Eukaryotic cell lines
☒	☐	Palaeontology and archaeology
☒	☐	Animals and other organisms
☐	☒	Clinical data
☒	☐	Dual use research of concern
☒	☐	Plants

Methods

n/a	Involved in the study	
☒	☐	ChIP-seq
☐	☒	Flow cytometry
☒	☐	MRI-based neuroimaging

Antibodies

Antibodies used

All antibodies used in this study are described in the appropriate subsection of the Methods section of the manuscript. Briefly, primary antibodies and lectins used in this study were: anti-A and -B(for reperfusion experiments; mouse mAb IgM; final titre in perfusate 1:128; Lorne Laboratories Ltd., Berkshire, UK); anti- blood group A antigen (1:150; mouse mAb IgM; HE-193; Invitrogen, Carlsbad, MA, USA); Ulex europaeus I lectin (1:50; anti-H; biotinylated; GTX01511; Genetex, Irvine, CA, United States); anti-C4d (1:100; rabbit mAb IgG; A24-T; ab136921; Abcam, Cambridge, UK); anti- C1qA (1:200; recombinant rabbit mAb IgG; EPR14634; ab189922; Abcam, Cambridge, UK); and, anti-C5b-9 (1:200; rabbit pAb IgG; A227; Complement Technology Inc., Tyler, TX, USA).
The secondary antibodies used were: anti-mouse IgM-AF555 (1:500; goat pAb IgG; A- 21426; Invitrogen, Carlsbad, MA, USA); anti-rabbit IgG-488 (1:750; goat pAb IgG; A28175; Invitrogen, Carlsbad, MA, USA); anti-mouse IgM-AF647 (1:2000; goat pAb IgG; A-21238; Invitrogen, Carlsbad, CA, USA) and, streptavidin-AF647 (1:2000; S32357; Invitrogen, Carlsbad, MA, USA).

Validation

Anti-blood group A antigen (1:150; mouse mAb IgM; HE-193; Invitrogen, Carlsbad, MA, USA) was validated for immunostaining of blood group A antigens on human kidney tissue (FFPE) on their website and in other human tissues in two further publications (doi: 10.1002/cam4.5476 and doi: 10.1016/s1499-3872(14)60010-2). Ulex europaeus I lectin (1:50; anti-H; biotinylated; GTX01511; Genetex, Irvine, CA, United States) has been validated extensively in the literature for binding H antigens, including in human tissue staining (see DOI: 10.1093/bjs/znac293 for kidney specific use). Validation of anti-A and-B (mouse mAb IgM; Lorne Laboratories Ltd., Berkshire, UK) binding to human blood group antigens on red blood cells was provided on the certificate of analysis, with titres determined via test tube, microplate, BioVue card and Diamed Card analysis with reagent red blood cells. The titre of concentrated antibody was determined via microplate agglutination analysis of blood group A and B 3% reagent red blood cells. All anti-blood group antigen antibodies and lectins were also validated internally for human kidney tissue immunostaining use prior to this study through staining of human kidney tissue of different blood groups, where no cross-reactivity of blood group staining was observed. Anti-C4d (1:100; rabbit mAb IgG; A24-T; ab136921) was validated by Abcam as described on their website, and through the literature (see DOI: 10.1097/CM9.0000000000001685). Anti- C1qA (1:200; recombinant rabbit mAb IgG; EPR14634; ab189922; Abcam, Cambridge, UK) was validated by Abcam for human kidney tissue FFPE immunostaining as described on their website (see doi: 10.1038/s42003-020-01298-1 for rat kidney immunofluorescence staining). Anti-C5b-9 (1:200; rabbit pAb IgG; A227; Complement Technology Inc., Tyler, TX, USA) was validated as described in the certificate of analysis. The manufacturer describes the sensitivity as monospecific for human SC5b-9 complex in plasma, with no reaction with non-activated normal human serum or plasma. This antibody reacts with cell surface-bound C5b-9 complex.

Secondary antibodies were validated for non-specific binding in no primary antibody staining controls included in all staining experiments. In all cases, no staining was observed in no primary antibody controls above a background level. Immunofluorescence images on the manufacturer's website also demonstrate the lack of focal staining in the absence of a primary antibody. Additional information on validation can be found on the Thermo Fisher Scientific (Invitrogen) website.

Clinical data

Policy information about clinical studies
All manuscripts should comply with the ICMJEguidelines for publication of clinical research and a completedCONSORT checklist must be included with all submissions.

Clinical trial registration

N/A

Study protocol

N/A

Data collection

Data pertaining to organ donor demographics was obtained from NHSBT between July 2022 and May 2023. All donor data was anonymised to the investigators prior to data collection.

Outcomes

Only clinical data pertaining to deceased organ donor demographics was collected for this study. No clinical outcomes were measured in this study.

Plants

Seed stocks

N/A

Novel plant genotypes

NA

Authentication

N/A

Flow Cytometry

Plots

Confirm that:

- [x] The axis labels state the marker and fluorochrome used (e.g. CD4-FITC).
- [x] The axis scales are clearly visible. Include numbers along axes only for bottom left plot of group (a 'group' is an analysis of identical markers).
- [x] All plots are contour plots with outliers or pseudocolor plots.
- [x] A numerical value for number of cells or percentage (with statistics) is provided.

Methodology

Sample preparation	Human blood group A RBCs were fixed in 0.05% glutaraldehyde for 10mins, then centrifuged at 600g for 5 mins. RBCs were washed twice in PBS and then diluted to 1% Hct. For flow staining, 50ul of perfusate was incubated with 50ul 1% RBCs for 30mins at RT. RBCs were washed 3x in PBS + 0.6% BSA and then resuspended in 100ul PBS + 0.6% BSA with goat anti-ms IgM-647 (A-21238; Invitrogen, Carlsbad, CA, USA) for 30mins at RT in the dark. RBCs were washed three times then resuspended in 250ul PBS + 0.6% BSA for flow cytometry.
Instrument	Samples in 96-well plates were analysed using an NXT Attune with Autosampler (Thermo Fisher Scientific, Waltham, Massachusetts, United States).
Software	FlowJo 10.9.0 (Ashland, Oregon, United States) was used to analyse all flow cytometry data.
Cell population abundance	All samples were prepared from 1% RBCs derived from packed red blood cells and all RBCs gated were used for flow cytometric assessment.
Gating strategy	Purified 1% RBCs obtained from packed red blood cells were used for all experiments. The RBC gate was determined on a graph of FSC-A/SSC-A, where debris was excluded as described in the Supplementary Information. Singlets were determined based on a graph of FSC-A/FSC-H. The MFI of all single cells excited with RL1-A (anti-ms IgM-AF647) was recorded. Positive populations were determined based on the RL1-A fluorescence from RBCs incubated with antibody at the titre included in reperfusion experiments. Negative populations were determined with RBCs incubated with perfusate samples from before antibody addition and subsequently stained with anti-ms IgM-AF647.

- [x] Tick this box to confirm that a figure exemplifying the gating strategy is provided in the Supplementary Information.
